# Newly Diagnosed Children with Cancer Have Lower 25-Vitamin D Levels than Their Cancer-Free Peers: A Comparison across Age, Race, and Sex

**DOI:** 10.3390/cancers14102378

**Published:** 2022-05-12

**Authors:** Michell Fullmer, Annelise Su, Steven Bachrach, Jobayer Hossain, Heidi H. Kecskemethy

**Affiliations:** 1 Nemours Center for Cancer and Blood Disorders, Nemours Children’s Health, Wilmington, DE 19803, USA; michell.fullmer@nemours.org; 2 University of Richmond, Richmond, VA 23173, USA; annelise.su@richmond.edu; 3 Department of Pediatrics, Nemours Children’s Health, Wilmington, DE 19803, USA; steven.bachrach@nemours.org; 4 Nemours Biomedical Research, Nemours Children’s Health, Wilmington, DE 19803, USA; jhossain@nemours.org; 5 Department of Radiology, Nemours Children’s Health, Wilmington, DE 19803, USA

**Keywords:** 25(OH)D, cancer, child, deficiency, diagnosis, pediatrics, vitamin D

## Abstract

**Simple Summary:**

In this cross-sectional retrospective review of serum 25(OH)D levels in 544 children, children newly diagnosed with cancer (*n* = 136) had significantly lower 25(OH)D levels at diagnosis than their age-, sex-, and race-matched peers without cancer from the same geographic region of the US (*n* = 408). Significant differences were evident: older children exhibited lower 25(OH)D levels, children of color displayed higher levels of insufficiency, and black children were most deficient.

**Abstract:**

Children with cancer have a greater risk for vitamin D concerns because of compromised health before diagnosis, the disease itself, and treatments for the cancer. This IRB-approved retrospective, matched case–control study of children with and without cancer included three race categories: black, other, and Caucasian. This is the first study to directly compare serum 25-hydroxy vitamin D (25(OH)D) levels and status in newly diagnosed pediatric cancer patients with age-, sex-, and race-matched cancer-free children from the same geographic region of the US, all of whom are free from other conditions that negatively impact 25(OH)D levels. Univariable and multivariable ordinal logistic regressions were performed. In the 544 children (mean age of 8.5 years, 53% female), there were 136 newly diagnosed children with cancer and 408 matched non-cancer controls. Serum 25(OH)D levels at cancer diagnosis were lower (22.4 ng/mL) than in controls (30.1 ng/mL; *p* < 0.0001). Differences persisted across race (*p* < 0.001) and age (*p* < 0.001), but not sex. Older children exhibited lower 25(OH)D levels. Only 18.4% of the children with cancer had sufficient levels. Black children with cancer had the greatest rate of deficiency (39%). Race differences were evident: children of color (other and black) displayed higher levels of insufficiency; black children were most deficient.

## 1. Introduction

Vitamin D deficiency is a health concern for children worldwide [1]. Vitamin D insufficiency has been reported in 42% to 61% of healthy US adolescents [2,3], and, over the past several decades, vitamin D levels have decreased in healthy US children [4]. Suggested reasons for this trend include less time spent outdoors, reduced intake of vitamin-D-fortified foods, and increased use of sunscreen [4,5]. The classification of vitamin D levels and recommendations has also changed over the years, and classification of deficient, insufficient, and sufficient levels currently varies between professional organizations [6,7]. 

The role of vitamin D in health and the prevention of disease has been increasingly recognized, with particular interest in its role in cancer [8,9,10,11,12]. Vitamin D has broad effects within the body, having a role in immune function and cellular processes including proliferation, differentiation, apoptosis, and angiogenesis [13,14,15,16]. Vitamin D receptors are expressed in most cells in the body and have been shown in cancer cells [17]. Some cancers, such as breast and lung cancer, have mutated genes that cause an under-expression of vitamin D receptors, resulting in weakened vitamin D signaling pathways [18]. Vitamin D may have a protective effect against several adult cancers including colon, breast, prostate, colorectal, and ovarian [11], and a decreased all-cancer risk has been reported with vitamin D supplementation [19]. Our understanding of the role of vitamin D in both adult and pediatric cancer continues to evolve. 

Children with cancer are at greater risk for a compromised vitamin D status than the general population because of compromised health prior to diagnosis, the disease itself, and treatments for the cancer [10,16,20,21]. Compromised vitamin D status has a deleterious effect on bone mineral density (BMD), a known effect of cancer therapy for childhood cancer survivors [10,22,23,24,25]. Lower overall 1-year survival rates have been reported in children who had stem cell transplants and who had poor vitamin D levels [26]. 

While vitamin D status in children with many types of cancer has been examined in numerous studies, vitamin D status in children at the time of diagnosis has only recently been described [16,27,28,29]. Study designs, sample sizes, and measurement parameters of these reports vary, and none utilized a matched case–control study design. Furthermore, the other studies did not specifically exclude comorbidities that might negatively impact vitamin D status. 

In this study, we (1) describe vitamin D status at the time of pediatric cancer diagnosis by examining serum 25-hydroxy vitamin D (25(OH)D) levels in newly diagnosed children with cancer and compare them with 25(OH)D levels from a group of age-, race-, and sex-matched cancer-free peers from the same geographic region of the US, and (2) examine vitamin D status by age, sex, and race within and across the groups. 

## 2. Materials and Methods

The study design was a retrospective, matched case–control study of children with newly diagnosed cancer and children without cancer, using clinically obtained information. The study was conducted according to the guidelines of the Declaration of Helsinki and approved by the Nemours Institutional Review Board (protocol 802368, approved 10/30/15). All data were extracted from the electronic medical records of children treated at our pediatric tertiary-care center located in the mid-Atlantic region of the United States (latitude 39.7 degrees north) between January 2012 and January 2018. Data were retrieved through a data warehouse search of our EMR using ICD-9 codes for pediatric cancers seen at our institution. Table 1 lists the included cancer diagnoses.

### 2.1. Study Population 

All children had serum 25(OH)D levels drawn as part of clinical care. Those with oncology diagnoses had 25(OH)D levels drawn within 2 months of diagnosis. A control group of children without cancer who were age-, race-, and sex-matched with the children with cancer were identified from the same institution and time period. Each oncology patient was age-, race-, and sex-matched with three non-oncology patients. The two groups had a similar seasonal distribution of 25(OH)D results.

Exclusions for both groups included the following: (1) medication use that might affect vitamin D status (phenytoin, phenobarbital, carbamazepine, primidone, rifampin, chronic laxative use, cholestyramine, and corticosteroids); (2) history of diagnoses that may influence vitamin D status, including gastrointestinal, malabsorptive, chronic inflammatory, renal, or hepatic conditions (Table 2); and (3) vitamin D supplementation within 6 months prior to the serum 25(OH)D level.

### 2.2. Data

Data collected included age, sex, race, diagnoses/medical history, and current medications. Race was defined as Caucasian, black, or “other” (non-white Hispanic, Asian, and Native American). All 25(OH)D levels were processed in the same laboratory, utilizing the same assay, and reflected in ng/mL. The Endocrine Society recommendations were used to classify vitamin D status [6].

Data were grouped by patient type: oncology versus control group; then by race, sex, and age; and by classification of serum 25(OH)D levels.

### 2.3. Data Analysis

Study variables were summarized first by patient type (oncology **vs.** control), then by race, sex, and age. Levels of 25(OH)D and the ordinal categories of deficient, insufficient, and sufficient (Endocrine Society classifications) [6] were summarized by patient type and characteristic (race, sex, and age). The mean and standard deviation were calculated and used to summarize the continuous variables and counts, and percentages were used for categorical variables. Two-sample t-test, one-way analysis of variance (ANOVA), simple regression, the chi-square test, and univariable ordinal logistic regression were used, as appropriate, to assess the association (unadjusted) between the outcome (25(OH)D as a continuous and ordinal variable) and patient types and characteristics. An analysis of covariance (ANCOVA) was used to compare the mean 25(OH)D between patient types after controlling for race, sex, and age. Because the interaction of race and patient type was highly significant, we also stratified the ANCOVA by race. An ordinal logistic regression was used to determine the association of patient types with 25(OH)D deficiency. Model assumptions were checked before analysis. All tests were two-tailed with a level of significance of 0.05. The statistical software packages SPSS version 22 (IBM, Armonk, NY, USA) and R version 3.5.2 were used for data analysis.

## 3. Results

A total of 544 children were included in the study: 136 with newly diagnosed cancer (68 female) and 408 children in the age-, race-, and sex-matched control group. The mean ages were 8.5 years for the oncology group and 8.4 years for the control group (Table 3). Ages ranged from 1 month to 19.8 years. The group comprised 62% Caucasian, 18% black, and 19% “other” races (Table 4). There were no differences in race, sex, or age between the oncology and control groups (Table 5). As a whole, age was correlated with 25(OH)D levels (*p* < 0.001).

### 3.1. Within-Group Comparisons

Differences in serum 25(OH)D were seen between Caucasian and black children (*p* < 0.0001), and Caucasian and “other” (*p* = 0.001), but not between black children and children of “other” races (*p* = 0.896). There were no differences seen in mean 25(OH)D levels between males and females overall (*p* = 0.422), and there were no significant differences in 25(OH)D levels between males and females within each racial category: Caucasian (*p* = 0.036), black (*p* = 0.458), and “other” (*p* = 0.495).

### 3.2. Oncology versus Control 25(OH)D Levels by Variable

**Figure 1:**
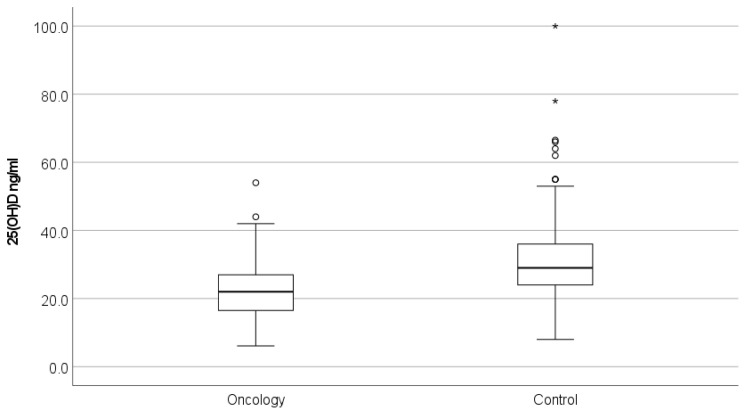
Serum 25(OH)D levels were significantly lower (*p* < 0.0001) in children newly diagnosed with cancer than in children without cancer. * *p* < 0.05.

**Figure 2:**
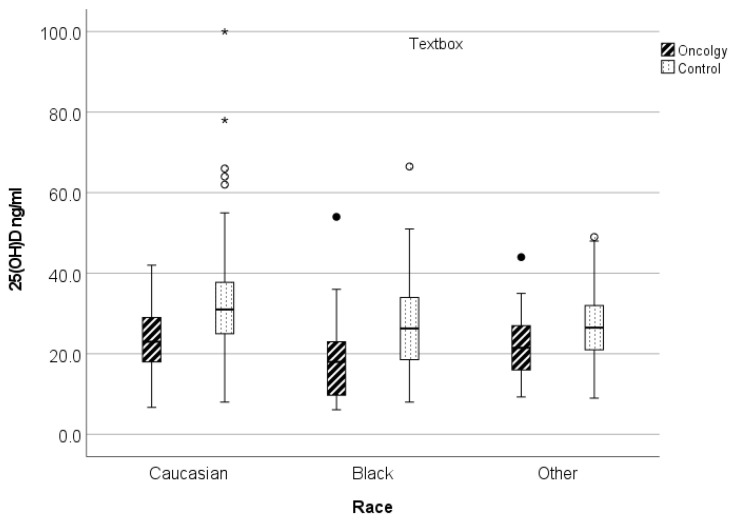
Mean serum 25(OH)D levels differ between children with and without cancer and between races, with Caucasians having the highest levels. * *p* < 0.05.

Average serum 25(OH)D levels were significantly lower in the newly diagnosed oncology patients compared with the matched control group, with mean values of 22.4 (±8.7) ng/mL and 30.1 (±10.7) ng/mL, respectively (*p* < 0.0001) (Figure 1, Table 3). Significant differences in 25(OH)D levels were seen by age (*p* < 0.0001) and across race (*p* < 0.005), but not by sex, in the children with cancer compared to the children without cancer. Of the children with cancer, black children had the lowest serum 25(OH)D levels (18.4 ng/mL), followed by children in the “other” race category (21.9 ng/mL); Caucasian children had the highest levels (23.8 ng/mL) (Figure 2, Table 3). The highest levels of vitamin D for all children were observed in the Caucasian non-oncology control group (31.8 ng/mL). Based on regression analyses, as age increased, 25(OH)D levels decreased. Because the interaction of race and patient type was highly significant, we also stratified the ANCOVA by race. After adjusting for race, the difference in serum 25(OH)D levels between the oncology and control groups persisted for race and age (Table 5).

### 3.3. Classification of 25(OH)D Status

As per the Endocrine Society recommendations, serum 25(OH) vitamin D level classifications are deficient (<20 ng/mL), insufficient (20–29 ng/mL), and sufficient (30–100 ng/mL) [6]. Of all the children with cancer, 18% had a sufficient level of 25(OH)D, while 49% of non-oncology controls had a sufficient level; 82% of the children with cancer were either deficient or insufficient, with 40% being deficient (Table 5). The control group demonstrated a 52% deficient or insufficient level, with 15% being deficient (Table 5).

When examining by race, Caucasian children had the greatest level of 25(OH)D sufficiency (47.1%) (Table 5) and the highest mean levels of 25(OH)D in both the cancer and control groups (Table 3). Black children had the highest level of deficiency, occurring in 39% of the children, compared with 23% of “other” race children and 15% of Caucasian children (Table 5). “Other” race children had the greatest rate of insufficiency (48.1%). The children with the greatest incidence of below sufficient levels of 25(OH)D were “other” race children (71.2%), followed by black children (67%) and Caucasians (52.9%) (Table 5).

## 4. Discussion

In this study, we found that children with newly diagnosed cancer had significantly lower 25(OH)D levels compared with a group of age-, sex-, and race-matched children without cancer from the same geographic region of the US and sampled with the same seasonality of measures. This study is one of the few that has focused on vitamin D status at the time of an oncology diagnosis and is unique because of the matched case–control study design where each oncology patient was matched with three non-oncology patients for age, race, and sex, adding to the body of evidence describing compromised vitamin D status in children newly diagnosed with cancer. Because this was a retrospective study utilizing a convenience sample of clinical patients, our control group consisted of patients who had their serum vitamin D checked clinically. Therefore, there was likely a concern about vitamin D status, so this group may have been at greater risk for suboptimal vitamin D levels. Despite this possibility, the average 25(OH)D level for the control group was 30.1 ng/mL, which is above the average of 27.24 ng/mL reported in US children from the National Health and Nutrition Examination Survey (NHANES) [30]. The differences in serum 25(OH)D between the children with cancer and those without persisted across race and age, but not sex. After multivariable and univariable analyses, the differences between those with and without an oncology diagnosis persisted.

The average 25(OH)D level of 22.5 (±8.7) ng/mL in the newly diagnosed children with cancer in this study was low, similar to the reported average values by Aristizabal et al. [29] (27.5 [+/−12.1] ng/mL) and Genc et al. [27] (16.75 ng/mL). Differences in 25(OH)D levels by sex were not observed in our study, which is in line with similar findings in multiple studies evaluating vitamin D levels in children newly diagnosed with cancer [27,28,31], and this was also true when sex was evaluated by race in our study. Significantly different 25(OH)D levels by race were seen in our study, which was expected considering that differences in vitamin D status by race and ethnicity have been described both in large population studies [3,4,32] and in children with newly diagnosed cancer [29,33]. Various factors, including socioeconomic or biologic, or both, may contribute to these differences. One recent study reported significantly lower serum 25(OH)D levels in children who were Hispanic compared with non-Hispanic whites [29]. We did not assess ethnicity in our study. Because race had such a strong interaction in our analyses, we included race in our model for multivariable analyses. After univariable and multivariable analyses, the differences in race persisted, with significant differences in vitamin D levels between Caucasian and black children, and Caucasian and “other” race children. There were no differences observed between black and “other” race children, possibly because of the small sample sizes in those groups. 

On the basis of regression analyses, we saw an inverse relationship between age and serum 25(OH)D levels: as age increased, serum vitamin D levels decreased. An age effect has been described in the general US population [32] and in other studies of children who are newly diagnosed with cancer [16,29,31,34,35] whereby younger children have higher serum 25(OH)D levels and older children are at greater risk for vitamin D deficiency. This may be partly due to changes in dietary choices and differing sun exposure related to how time is spent and the style of dress as children mature [36,37].

Reports of incidence of vitamin D deficiency and insufficiency in children with cancer use differing standards for classification; the Institute of Medicine standards vary from the Endocrine Society because they were created for different purposes. However, the application of these various standards can make a direct comparison of challenging reports. Furthermore, 25(OH)D levels in healthy populations vary around the globe and are influenced by nutrient fortification practices of countries, availability of food supply, regional and religious food practices, skin pigmentation and sun exposure due to latitude, use of sunscreen, and geographic and religious clothing practices that limit skin exposure to UV rays.

We found that 40% of the newly diagnosed children with cancer had serum 25(OH)D levels at the deficient level (<20 ng/mL), as per the Endocrine Society guidelines, and 42% were insufficient (20–29 ng/mL). This finding is similar to that of Helou and colleagues [31], who reported serum 25(OH)D levels of less than 20 ng/mL in 43% of the 89 children at a similar latitude (37° N) to our study (39.7° N) in the eastern United States and with a similar proportion of boys and girls (42% female vs. 43% female), though the racial demographics differed. Similarly, in another US study of 163 children who had serum 25(OH)D levels checked at the time of cancer diagnosis, 32% of the children were deficient [29].

Outside the US, reported rates of 25(OH)D deficiency in children at the time of a cancer diagnosis are higher, which is reflective of global rates of vitamin D deficiency [38]. In a Turkish study of 86 children with a new cancer diagnosis, 63% were found to be 25(OH)D deficient (<20 ng/mL) [27]. Jackmann et al. [35] used different classification levels for serum 25(OH)D for the 295 Swedish children whose 25(OH)D level was checked at cancer diagnosis, but reclassification as per the Endocrine Society guidelines produced a 72.9% deficiency rate. This higher rate than that observed in our study is in agreement with a report of 42 children in Turkey in which 79% were deficient [28]. This higher level of vitamin D deficiency was described in a systematic review and meta-analysis examining the prevalence and possible causes of vitamin D deficiency in children with cancer, in which the authors note a higher prevalence of vitamin D deficiency and insufficiency in Europe compared with the US [16].

A limitation of this study is the retrospective design, which did not allow for the evaluation of the overall nutritional status of the subjects. Furthermore, we were unable to assess vitamin or mineral supplementation, or both, beyond what was recorded in the electronic medical records. Particularly within the newly diagnosed children with cancer, variable nutritional status might have been contributory to the greater number of children with suboptimal levels of serum 25(OH)D compared to the non-oncology controls. We did not approach our analysis of these data from a socioeconomic perspective or by grouping the data by specific types of cancers including solid tumors versus leukemia/lymphoma. These factors are additional areas for further exploration. Our study is limited to the mid-Atlantic region of the US and is therefore not representative of all children or the general US population of children. We were not able to evaluate the overall medical state of the subjects at the time of the 25(OH)D assessment. We limited participants to those who had vitamin D levels drawn within 2 months of diagnosis; the majority (85%) had vitamin D levels drawn within 1 month of diagnosis. We also excluded patients with known or suspected medical conditions and those taking medications that might affect serum 25(OH)D levels. It is possible that these children had other unknown or undiagnosed problems that could have affected their vitamin D status. Finally, our study population included varying demographics, which yielded limited sample sizes for non-Caucasian children.

This study shows that children with cancer of all races demonstrated significantly lower serum 25(OH)D levels at diagnosis than their age-, sex-, and race-matched non-oncology peers. Patients of color were at greater risk for vitamin D insufficiency, with black children exhibiting the greatest risk. The significance of this finding relative to the development or progression of cancer is unclear, but worthy of further investigation.

## 5. Conclusions

This paper adds to the increasing evidence that children newly diagnosed with cancer are at greater risk for a compromised vitamin D status, and it underscores the importance of prompt evaluation of 25(OH)D levels when a child is diagnosed with cancer. Because of these findings, and other similar studies, institutions caring for children with cancer are beginning to consider and evaluate the implementation of vitamin D testing at the time of diagnosis as a part of standard care [39]. Within our own pediatric health care system, we have observed varying practices regarding vitamin D testing at the time of cancer diagnosis in children. The implementation of a care pathway, including serum 25(OH)D testing upon a cancer diagnosis, will allow for the prompt identification of patients who require a correction of vitamin D deficiency or insufficiency to improve patient health and response to treatment, and to help prevent the known bone health problems that children with cancer often develop. 

This study shows that children with cancer of all races demonstrated significantly lower 25(OH)D levels at diagnosis than their age-, sex-, and race-matched non-oncology peers. Risk factors for compromised 25(OH)D status were older age and cancer diagnosis. Patients of color exhibited a greater incidence of vitamin D insufficiency, and black children had the greatest levels of deficiency. The significance of this finding relative to the development or progression of cancer is unclear, but worthy of further investigation.

## Figures and Tables

**Table 1:** Cancer diagnoses included in data warehouse search by ICD-9 codes.

Included Cancers
Acute lymphocytic leukemia Acute myeloid leukemia
Neuroblastoma
Osteosarcoma
Ewing’s sarcoma
Medulloblastoma
Rhabdomyosarcoma
Glioblastoma
Lymphomas (all)
Wilms tumor
Germ cell tumors
Atypical teratoid/rhabdoid tumor
Hepatocellular carcinoma
Yolk sac tumor
Embryonal carcinoma

**Table 2:** Diagnoses excluded from study.

Excluded Diagnoses	
Anorexia nervosa Aplastic anemia	Jejunostomy tube Juvenile idiopathic arthritis
Bulimia	Juvenile rheumatoid arthritis
Cerebral palsy	Kidney transplant
Chronic arthritis	Laparoscopic gastric band
Chronic renal disease	Liver transplant
Colostomy	Malabsorption
Crohn’s disease	Metabolic disorders
Cystic fibrosis	Morbid obesity
Gastric sleeve	Necrotizing enterocolitis
Gastroschisis	Osteogenesis imperfecta
Gastrostomy tube	Short gut syndrome
Grave’s disease	Sickle cell anemia
Hypophosphatemic rickets	Systemic lupus erythematosus
Ileostomy	Thalassemia
Inflammatory bowel disease	Ulcerative colitis

**Table 3:** Serum 25(OH)D levels in children with and without cancer, grouped by race and sex.

	Oncology					Control				
Grouping	*n*	Mean Age (yrs)	Range	Mean 25(OH)D (ng/mL)	SD	*n*	Mean Age (yrs)	Range	Mean 25(OH)D (ng/mL)	SD
Black (all)	25	9.4	0.5–17.9	18.4	10.8	75	9.3	0.5–17.2	27.6	11.8
Female	11	8.2	0.5–16.3	18.4	8.3	33	8.3	0.5–16.8	27.7	13.0
Male	14	10.3	3.0–17.9	18.4	12.8	42	10	2.0–17.2	27.5	10.9
Other (all)	26	8.3	1.4–16.3	21.9	8.2	78	8.2	1.3–16.5	26.9	8.3
Female	8	6.3	1.4–13.6	22.5	7.4	24	6.2	1.3–13.5	28.1	8.3
Male	18	9.2	2.3–16.3	21.6	8.7	54	9.1	2.0–16.5	26.4	5.1
Caucasian (all)	85	8.3	0.6–19.2	23.8	7.8	255	8.2	0.1–19.8	31.8	10.7
Female	39	8.5	0.6–17.3	24.4	7.9	117	8.4	0.1–17.8	33.0	11.7
Male	46	8.1	0.5–19.2	23.3	7.8	138	8.0	0.03–19.8	30.7	9.8
Total	136	8.5	0.5–19.2	22.4	8.7	408	8.4	0.03–19.8	30.1	10.7

yrs = years; 25(OH)D = 25-hydroxy vitamin D; SD = standard deviation.

**Table 4:** No significant differences exist in the categorical variables of race, sex, and age between the children with and without cancer.

Variable	Oncology	Control	Overall	*p*-Value
	(*n* = 136)	(*n* = 408)	(*n* = 544)	
Race				>0.99
Caucasian	85 (62.5%)	255 (62.5%)	340 (62.5%)	
Black	25 (18.4%)	75 (18.4%)	100 (18.4%)	
Other	26 (19.1%)	78 (19.1%)	104 (19.1%)	
Sex				>0.99
Female	58 (42.6%)	174 (42.6%)	232 (42.6%)	
Male	78 (57.4%)	234 (57.4%)	312 (57.4%)	
Age				0.8
Mean (SD)	8.50 (4.98)	8.38 (4.93)	8.41 (4.94)	
Median [min, max]	8.33 [0.493, 19.2]	8.08 [0.0329, 19.8]	8.10 [0.0329, 19.8]	

**Table 5:** 25(OH)D classification and univariable and multivariable ordinal logistic regressions to determine the association of 25(OH)D levels with predictors.

Variable		Overall (%)	25(OH)D Classification			Univariable		Multivariable	
			Deficient (%)	Insufficient (%)	Sufficient (%)				
		*n* = 544	*n* = 113	*n* = 208	*n* = 223	POR (95% CI)	*p*-Value	Adjusted POR (95% CI)	*p*-Value
Group	Oncology	136 (25)	54 (39.7)	57 (41.9)	25 (18.4)	0.25 (0.17, 0.36)	<0.001	0.22 (0.15, 0.32)	<0.001
	Control	408 (75)	59 (14.5)	151 (37.0)	198 (48.5)	ref		ref	
Race	Caucasian	340 (62.5)	50 (14.7)	130 (38.2)	160 (47.1)	ref		ref	
	Black	100 (18.4)	39 (39.0)	28 (28.0)	33 (33.0)	0.39 (0.25, 0.59)	<0.001	0.40 (0.26, 0.62)	<0.001
	Other	104 (19.1)	24 (23.1)	50 (48.1)	30 (28.8)	0.52(0.34, 0.78)	0.002	0.49 (0.32, 0.75)	0.001
Sex	Female	232 (42.6)	43 (18.5)	84 (36.2)	105 (45.3)	1.33 (0.97, 1.83)	0.078	1.24 (0.89, 1.74)	0.204
	Male	312 (57.4)	70 (22.4)	124 (39.7)	118 (37.8)	ref		ref	
Age	mean (SEM)	8.41 (0.21)	10.50 (0.45)	8.90 (0.34)	6.90 (0.31)	0.89 (0.87, 0.92)	<0.001	0.89 (0.86, 0.92)	<0.001

25(OH)D = 25-hydroxy vitamin D; POR = proportional odds ratio; CI = confidence interval; ref = reference; SEM = standard error of the mean.

## Data Availability

The data presented in this study are available on request from the corresponding author.
